# Expression of the senescence marker p16^INK4a^ in skin biopsies of acute lymphoblastic leukemia survivors: a pilot study

**DOI:** 10.1186/1748-717X-8-252

**Published:** 2013-10-31

**Authors:** Sophie Marcoux, Oanh NL Le, Chloé Langlois-Pelletier, Caroline Laverdière, Afshin Hatami, Philippe Robaey, Christian M Beauséjour

**Affiliations:** 1 Centre de recherche du CHU Sainte-Justine, Montréal, Québec, Canada; 2 Faculté de médecine, Université de Montréal, Montréal, Québec, Canada; 3 Département de pharmacologie, Université de Montréal, Montréal, Québec, Canada; 4 CHU Sainte-Justine, 3175 Chemin de la Côte-Ste-Catherine, Montréal H3T 1C5, Québec, Canada

**Keywords:** Ionizing radiation, Cellular senescence, p16^INK4a^ expression, Childhood acute lymphoblastic leukemia, Cancer treatment long-term sequelae

## Abstract

**Background:**

Most childhood cancer survivors will develop ionizing radiation treatment-related health conditions that, in many instances, resemble age-associated pathologies. Treatment-induced premature senescence could be an underlying mechanism.

**Findings:**

Here we wanted to know whether the expression of p16^INK4a^, a senescence/aging biomarker, is increased in skin biopsies of acute lymphoblastic leukemia survivors (ALL), previously exposed to chemotherapy and radiation therapy. Several years post-treatments, we found p16^INK4a^ mRNA levels are 5.8 times higher in scalp skin biopsies (targeted by cranial irradiation therapy) compared to buttocks skin biopsies (n = 10, p = 0.01).

**Conclusions:**

These results demonstrate for the first time that premature senescence is induced in pediatric cancer survivors and that p16^INK4a^ expression could be used as a potential biomarker in this population.

## Findings

### Introduction

The majority of children treated for cancer will eventually develop at least one chronic medical condition related to their previous exposure to chemotherapy and/or radiation therapy [1,2]. Identifying a biomarker indicative of global damage level would be useful in predicting individual sensitivity to anti-cancer treatment long-term side effects.

Cellular senescence was first observed in vitro and for a long time, was believed exclusively dependent on telomeres length. Since then, it has been shown that cellular senescence can be triggered prematurely by various stimuli such as DNA damage, oncogenic stress and oxidative stress [3]. More specifically, ionizing radiation (IR) and chemotherapy can induce p16^INK4a^, a senescence marker, in murine models [4,5]. We also showed that a DNA damage response (a hallmark of senescence) is observed up to one year post-exposure to IR in the skin of cancer patients [5]. Induction of senescence has been shown to be triggered as a tumor preventive mechanism in humans [6] and, more recently, as a program limiting fibrosis in mice stellate liver cells [7].

p16^INK4a^ is a tumor suppressor gene and arguably the best biomarker for cellular senescence [8]. Briefly stated, it induces irreversible cell cycle arrest by preventing Rb phosphorylation [9,10]. Its expression correlates with aging in various mice tissues and human lymphocytes [11-13]. Expression of p16^INK4a^ was also shown to limit progenitor cells renewal with age in neural [14], bone marrow [15] and islet mouse tissues [16]. Moreover, clearance of p16^INK4a^ positive cells delays some aging-associated disorders [17]. Given these evidences, it is reasonable to hypothesize that an increase in p16^INK4a^ expression following exposure to IR and/or chemotherapy may contribute to treatment-related late effects observed in cancer survivors. This project aimed at verifying whether a higher expression of p16^INK4a^ could be found in patients’ skin areas directly targeted by radiation therapy.

## Materials and methods

The project was approved by the Institutional Review Board and informed consent was obtained from each participant. Patients were recruited among the long-term follow-up clinic. All patients were treated according to Dana Farber Cancer Institute ALL Consortium treatment protocols (91–01, 95–01 or 00–01) [18-20]. Eligibility criteria were: having received an ALL diagnosis before age 18, having no evidence of active disease, having received cranial irradiation as part of the ALL treatment, having no history of other previous disease requiring chemotherapy and/or radiation therapy. The first 10 families recruited were enrolled in this study (participation rate of approximately 30%).

### Skin biopsies

This procedure was performed by a dermatologist and consisted of three millimiters diameter biopsies containing all skin layers. For each participant, 2 biopsies were performed: one in the scalp region targeted by cranial radiation therapy (‘scalp’; n = 10), and one from a non-irradiated (n = 8) or irradiation-exposed in the context of total body irradiation (TBI) (n = 2) (‘buttocks’). Whenever possible, a skin biopsy from the buttocks of siblings was also obtained.

### p16^INK4a^ measurements

Biopsies were kept in an RNAlater solution (QIAGEN Cat#76160) at a temperature of 4°C. RNA extraction was done by using the RNeasy Lipid Tissue Mini kit (QIAGEN Cat#74804). Reverse transcription (RT) reactions were done by using the QuantiTect Reverse Transcription kit (QIAGEN). Expression of p16^INK4a^ mRNA was quantified by qPCR (standard curve method) using at least two independent RT reactions for each biopsy and the SensiMix TM SYBR Low ROX Kit (BIOLINE). The following primers were used: (forward) CCAACGCACCGAATAGTTACG, (reverse) GCGCTGCCCATCATCATG. 18 s expression was also measured, as a mean to normalize p16^INK4a^ levels. 18 s primers were (forward) TCAACTTTCGATGGTAGTCGCCGT, (reverse) TCCTTGGATGTGGTAGCCGTTTCT.

### Statistical analysis

Four measurements of p16^INK4a^ and 18 s mRNA were independently measured for each biopsy. The average of these measurements was used for statistical analyses. All statistical analyses were performed using SPSS 17.0.

## Results

**Figure 1:**
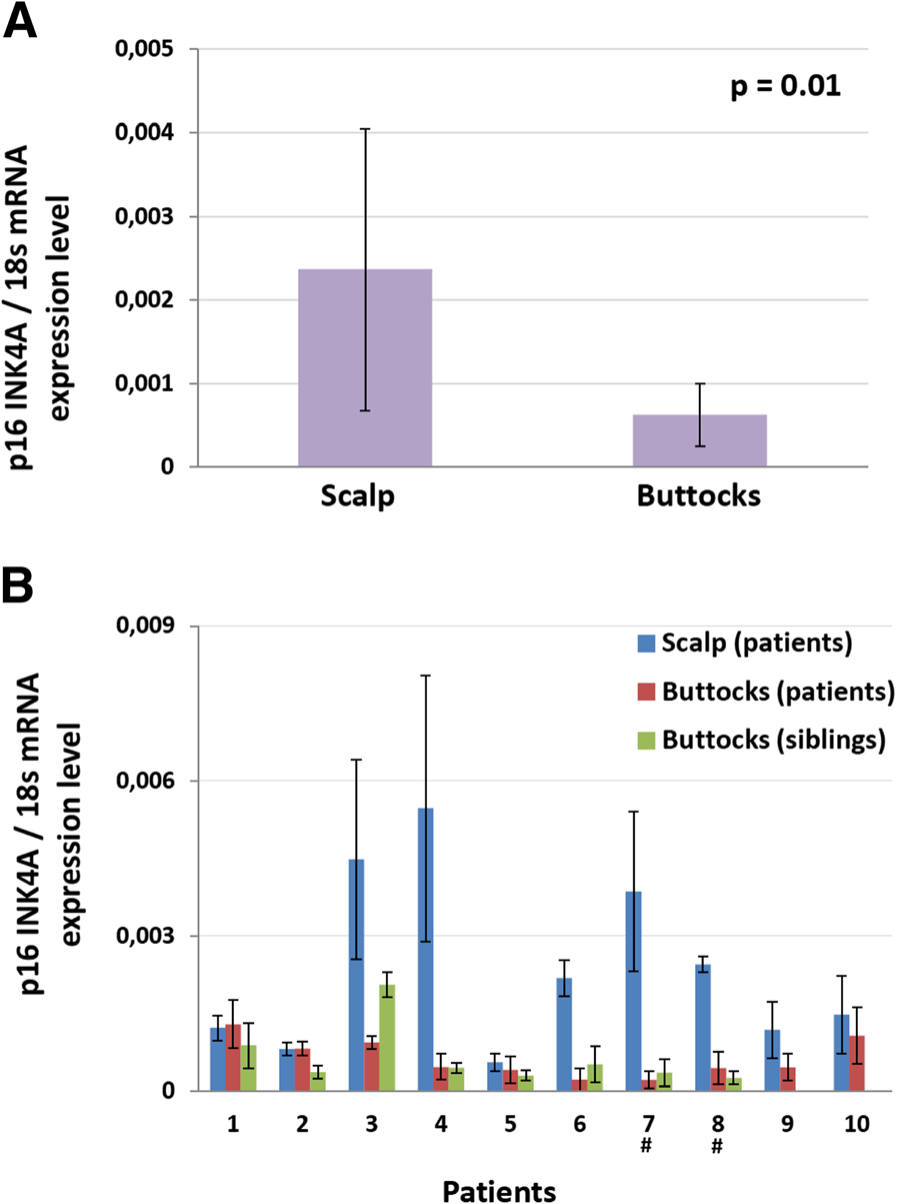
**p16**^**INK4A **^**mRNA levels in irradiated compared to non-irradiated tissues.** p16^INK4A^ mRNA was quantified by qPCR and was normalized using 18 s mRNA expression level. qPCR was run four times independently for each biopsy. Error bars represent +/− 1 S.D. **(A)** The average expression level for biopsies from patients’ scalp was found to be significantly higher than the average expression level for biopsies from the buttocks (n = 10 patients, paired t-test). **(B)** Individual participants’ p16^INK4A^ mRNA expression levels. # indicates patients who received 12 Gy TBI in addition to cranial irradiation. Skin biopsy from siblings was not available for patients 9 and 10.

Patients’ characteristics are shown in Table 1. A paired t-test revealed that p16^INK4a^ mRNA expression was 5.8 times higher in the scalp biopsies (‘irradiated zone’) compared to the buttock biopsies (‘non-irradiated zone’) (Figure 1A; p = 0.01). No significant differences in p16^INK4a^ expression levels were observed between buttock biopsies collected from patients compared to those collected from siblings (data not shown). This suggests that neither exposure to chemotherapy nor lower level of irradiation (2 patients had received total body irradiation in addition to cranial irradiation) leads to p16^INK4a^ expression in human skin. Of note, there is an important heterogeneity in the p16^INK4a^ fold increase expression in scalp biopsies (Figure 1B). Surprisingly, and in opposite to what we observed previously in irradiated mouse tissues [5], p21 and p14^ARF^ (a tumor suppressor gene expressed from the same locus as p16^INK4a^) were also generally increased in the scalp biopsies, although not reaching statistical significance (Additional file 1: Figure S1); p = 0.11 and p = 0.07 respectively. Yet, IL-6 mRNA levels (a senescence-associated secretory phenotype marker) [21] was not increased in all but one scalp biopsy (data not shown). Similarly, and in contrast to what was observed in benign prostatic hyperplasia [22], no increase in PML or PML-IV were observed.

**Table 1:** Patients’ characteristics

**Variables**	**Patients**									
	**1**	**2**	**3**	**4**	**5**	**6**	**7**	**8**	**9**	**10**
**Age at diagnosis**	5	7	3	5	10	9	3	1	4	3
Age biopsy	17	23	17	20	24	25	19	13	14	13
Gender^1^	F	M	M	M	M	M	F	M	M	M
Years since diagnosis	12	16	14	15	14	16	14	6	10	11
Treatment protocol DFCI	95-01	91-01	95-01	95-01	91-01	91-01	91-01	95-01	00-01	95-01
Stem cell transplantation	no	no	no	no	no	no	yes	yes	no	no
Cranial radiation dose (18 Gy)	yes	yes	yes	yes	yes	yes	yes	yes	yes	yes

^1^All patients were caucasian. F = female, M = male.

## Discussion

The senescence/aging biomarker p16^INK4a^ is more expressed in irradiated skin portions of ALL survivors compared to non-irradiated or scarcely irradiated portions. Importantly, it suggests that DNA damage-induced senescent cells in skin of these patients do not get cleared up, even after an average of 12 years post-diagnosis. We thus propose that p16^INK4a^ could be further studied as a potential biomarker for long-term sequelae in childhood ALL survivors and suggest that premature senescence could be considered as a mechanism behind side-effects in this population. Our finding that p14^ARF^ appears coregulated with p16^INK4a^ was unexpected based on our observation that it was not in irradiated mice tissues [5]. One explanation for this discrepancy, aside from the species difference, is the time post exposure which was much longer in patients. Noteworthy, the expression of both genes was shown to be coregulated during aging in rodents tissues [12], but not in human lymphocytes [13].

The small number of participants is a limitation of this pilot study. Despite the pain associated to the biopsy being estimated similar to that of taking a blood sample by participants, this method led to a significant number of potential participants to decline our invitation to the study. To ease patients’ recruitment, we measured p16^INK4a^ levels in blood lymphocytes, aiming for a sampling method more acceptable to participants. Unfortunately, no average difference in p16^INK4a^ levels was detected between a group of various childhood cancer survivors who had received radiotherapy as part of their treatment and a group of healthy participants (n = 11, data not shown). While Liu *et al.* have detected some differences in p16^INK4a^ levels with age using blood lymphocytes [23], our incapacity to do so could be explained by at least three possibilities: first, only a fraction of the progenitor lymphocytes are exposed during irradiation, given that the radiotherapy-targeted zone is restricted to the head. Second, the half-life of T lymphocytes is difficult to tell and it is very likely that a majority of those who were irradiated were cleared out by the time the blood samples were taken. Third, a lack of statistical power cannot be excluded. Another limitation is associated to the location of the biopsy. The upper occipital area was an indicated biopsy site given it is irradiated in childhood ALL patients as part of CNS prophylaxis. Buttocks were chosen as the internal control site for ethical reason, as it minimizes the visibility of the second scar. It is known that UV radiations can be genotoxic [24] and as such, it is possible that for some patients, extensive head sun exposure may have played a partial role in the elevated p16^INK4a^ expression observed compared to the biopsies from the buttock. Scalp biopsies were however taken from the upper occipital region, an area that is not expected to receive sun exposure unless it has been previously fully shaved, a haircut that none of the patient was bearing at the time biopsies were performed.

Using p16^INK4a^ expression level as a biomarker for long-term health problems in childhood cancer patients could be useful in the screening for patients at risk and in the development of optimized treatments. The heterogeneity in the sensitivity to treatment toxicity would be coherent with the heterogeneity in p16^IKN4A^ mRNA expression fold increase we observed (Figure 1B). Furthermore, since p16^INK4a^ is likely to be not only a biomarker, but also a mediator of senescence/aging, it may itself represent an interesting therapeutic avenue in the prevention of long-term health problems. Removal of senescent cells induced by radiotherapy in ALL survivors may be considered in the future to prevent long-term side-effects.

There is clearly a need for additional senescence studies using humans beings rather than murine models given the physiological differences reviewed in [25]. Comprehensive studies investigating the associations between senescence biomarkers, histological changes and organs impairment must be the next target. Individual factors behind the observed heterogeneity in long-term side-effects occurrence must also be further investigated: genetic studies, including polymorphisms studies, should provide important clues.

## Competing interests

The authors declare no competing financial interests.

## Authors’ contributions

Contribution: SM, CL, PR and CMB designed the experiments; SF, ONLL, CLP and AH performed the experiments and/or recruited patients; SM, CL and CMB analyzed the data and wrote the manuscript. All authors read and approved the final manuscript.

## Supplementary Material

Additional file 1: Figure S1 Marcoux et al. in pdf format containing a supplementary figure is also available. Click here for file
