# Supplementary material for: Expression of the senescence marker p16INK4a in skin biopsies of acute lymphoblastic leukemia survivors: a pilot study
Source: Radiat Oncol. 2013 Oct 31;8:252. doi: 10.1186/1748-717X-8-252 (PMC3827993; doi:10.1186/1748-717X-8-252)
Supplement: Additional file 1: Figure S1 — Marcoux et al. in pdf format containing a supplementary figure is also available. [file 1748-717X-8-252-S1.pdf]

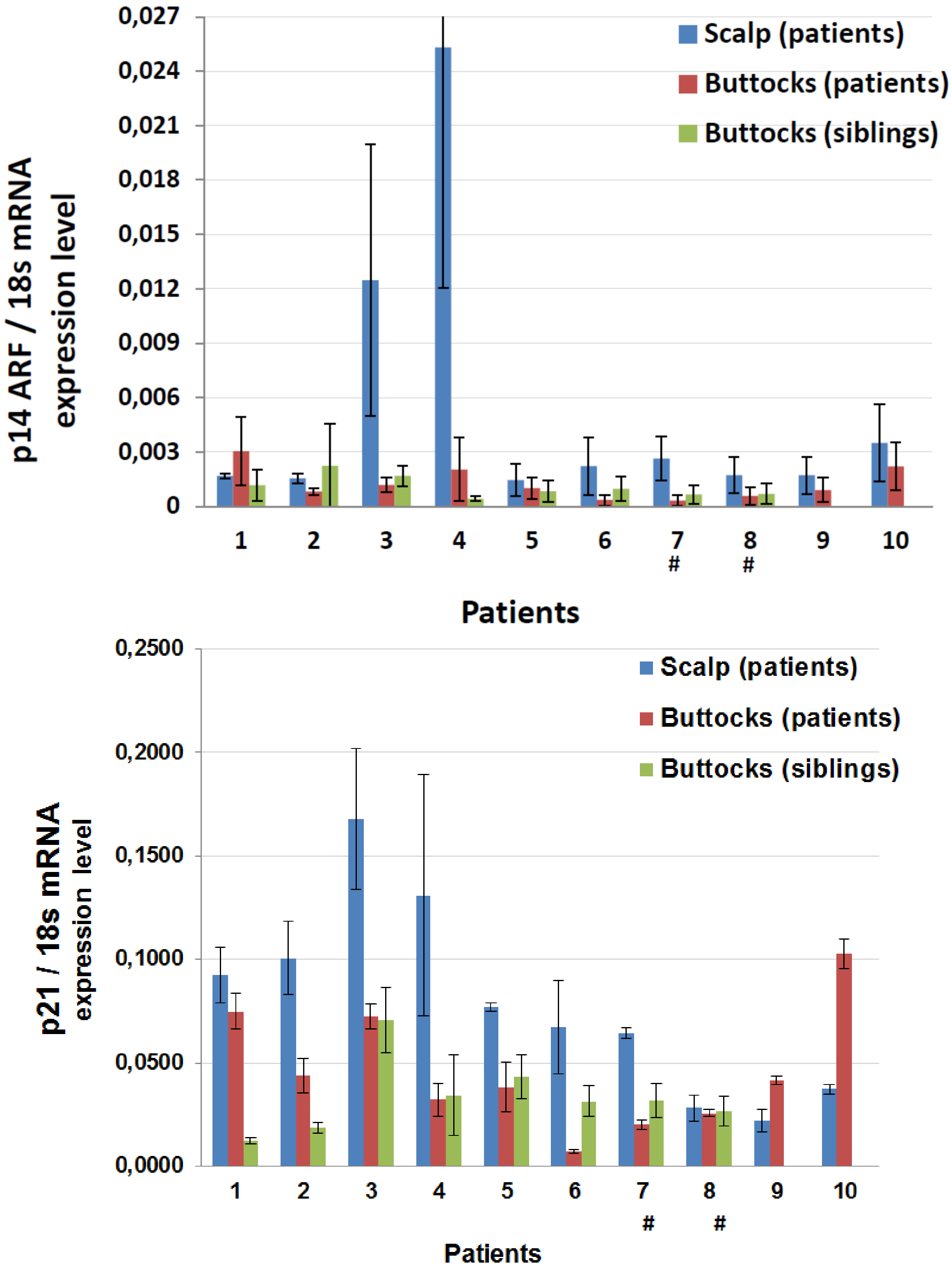

p14 ARF / 18s mRNA expression level
0,027
0,024
0,021
0,018
0,015
0,012
0,009
0,006
0,003
0
Scalp (patients)
Buttocks (patients)
Buttocks (siblings)
1
2
3
4
5
6
7 #
8 #
9
10
Patients
p21 / 18s mRNA expression level
0,2500
0,2000
0,1500
0,1000
0,0500
0,0000
Scalp (patients)
Buttocks (patients)
Buttocks (siblings)
1
2
3
4
5
6
7 #
8 #
9
10
Patients

FIGURE LEGEND

Figure S1: $p14^{ARF}$ and p21 mRNA levels in irradiated compared to non-irradiated tissues. $p14^{ARF}$ and p21 mRNA were quantified by qPCR and were normalized using 18s mRNA expression level. qPCR was run four times independently for each biopsy. Error bars represent +/- 1 S.D. The average expression level for biopsies from patients' scalp was not found to be significantly higher than the average expression level for biopsies from the buttocks (n = 10 patients, paired t-test, p = 0.11 and p = 0.07 for $p14^{ARF}$ and p21 respectively). Primers used were : human p21 forward 5'-ggcagaccagcatgacagatt-3'; reverse 5'-gcggattagggcttcctctt-3' and human p14ARF forward 5'-ctgaggagccagcgtctag- 3'; reverse 5'-cccatcatcatgacctggtcttcta-3'.
